# Deep Sequencing Analysis Reveals Distinctive Non-Coding RNAs When Comparing Tumor Multidrug-Resistant Cells and Extracellular Vesicles with Drug-Sensitive Counterparts

**DOI:** 10.3390/cancers12010200

**Published:** 2020-01-14

**Authors:** Diana Sousa, Rune Matthiesen, Raquel T. Lima, M. Helena Vasconcelos

**Affiliations:** 1 i3S-Instituto de Investigação e Inovação em Saúde, Universidade do Porto, 4200-135 Porto, Portugal; dsousa@ipatimup.pt (D.S.); rlima@ipatimup.pt (R.T.L.); 2 Cancer Drug Resistance Group, IPATIMUP—Institute of Molecular Pathology and Immunology of the University of Porto, 4200-135 Porto, Portugal; 3 Department of Biological Sciences, FFUP—Faculty of Pharmacy of the University of Porto, 4050-313 Porto, Portugal; 4 Computational and Experimental Biology Group, CEDOC, Chronic Diseases Research Centre, NOVA Medical School, Faculdade de Ciências Médicas, Universidade NOVA de Lisboa, Campo dos Mártires da Pátria, 130, 1169-056 Lisboa, Portugal; 5 Department of Pathology, FMUP—Faculty of Medicine of the University of Porto, 4200-319 Porto, Portugal; 6 Cancer Signalling & Metabolism Group, IPATIMUP—Institute of Molecular Pathology and Immunology of the University of Porto, 4200-135 Porto, Portugal

**Keywords:** Cancer, multidrug resistance, extracellular vesicles, next generation sequencing, small RNAs, microRNAs, pseudogenes

## Abstract

Multidrug resistance (MDR) is one of the main limitations of cancer treatment. The overexpression of drug-efflux pumps, such as P-glycoprotein (P-gp), is a major cause of MDR. Importantly, different studies have shown that extracellular vesicles (EVs) participate in the communication between MDR cells and drug-sensitive counterparts, promoting dissemination of the MDR phenotype. In the present work, we aimed to identify RNA species present in MDR cells and in EVs released by those cells, which may be associated with the MDR phenotype. The RNA content from two pairs (leukemia and lung cancer) of MDR (P-gp overexpressing) cells and their drug-sensitive counterparts, as well as from their EVs, was analyzed by deep sequencing. Our results showed distinctive transcripts for MDR cells and their EVs, when compared with their drug-sensitive counterparts. Remarkably, two pseudogenes (a novel pseudogene and RNA 5.8S ribosomal pseudogene 2) were found to be increased in EVs released by MDR cells in both leukemia and lung cancer models. Moreover, six miRs (miR-204-5p, miR-139-5p, miR-29c-5p, miR-551b-3p, miR-29b-2-5p, and miR-204-3p) exhibited altered levels in lung cancer MDR cells and their EVs. This study provides insights into the contribution of EVs to MDR.

## 1. Introduction

Cancer multidrug resistance (MDR) is a particular case of drug resistance, in which tumor cells fail to respond to more than one drug with different molecular structures and mechanisms of action [1,2]. MDR is the result of a network of altered cellular mechanisms, being one of the major challenges of cancer treatment. One of the most frequent causes of MDR is the overexpression of drug-efflux pumps such as P-glycoprotein (P-gp). Indeed, several studies have associated high levels of P-gp with drug resistance, particularly MDR, in a variety of tumor types [3]. Another important cellular alteration which was more recently described is the release of extracellular vesicles (EVs) by MDR cells [4].

EVs are small particles enclosed by a lipid bilayer which do not replicate, found in different biological fluids such as urine, blood, and saliva. EVs can be easily isolated from biological fluids or cell culture supernatants using different methodologies, such as ultracentrifugation, size exclusion chromatography, and affinity chromatography [5]. EVs are released and taken up by different types of cells, allowing horizontal communication between neighbors or distant cells [4]. Two main EV classes have been identified based on their biogenesis: exosomes and microvesicles. Typically, exosomes are developed by the endocytic pathway and microvesicles are produced by budding of the plasma membrane. In the past, these two classes of EVs were also distinguished on the basis of their size [6]. However, awareness of a size overlap between these two classes, especially in the smaller particle range (30–150 nm), is increasing and the recommendation of the International Society of Extracellular Vesicles (ISEV) is thus to name them collectively as “EVs” [7]. EVs’ cargo is comprised of fragments of DNA, RNAs, lipids, proteins, sugars, and metabolites [8]. Importantly, their rich heterogeneous cargo is protected from degradation caused by factors such as the ubiquitous extracellular RNases [9]. Remarkably, there is evidence that cancer cells release more EVs than normal cells [10], particularly after chemotherapy [11]. Therefore, EVs may represent an important source of biomarkers to be used in early diagnosis, prognosis, and evaluation of therapy responses in cancer [12,13].

The description of the horizontal transfer of RNAs, particularly microRNAs (miRs), as a mechanism of genetic exchange between cells mediated by EVs, was a breakthrough in the field [14]. Since then, many studies have been conducted, particularly in the cancer MDR context. Individual small RNAs or panels of small RNAs (particularly miRs) and long non-coding RNAs have been found in EVs and described to be responsible, at least in part, for the MDR phenotype in recipient cells [4]. They have been described to have a role in senescence suppression [15], promotion of cell invasion [16], and migration [17]. However, the cellular and molecular mechanisms underlying MDR dissemination triggered by non-coding RNAs associated with EVs (i.e., present in their cargo) have not been fully understood yet [18].

In the present work, we aimed to (i) confirm if the horizontal transfer of a drug-resistant phenotype mediated by EVs is possible from MDR non-small cell lung cancer (NSCLC) cells to their drug-sensitive counterparts; (ii) identify RNA species putatively associated with the MDR phenotype, in an NSCLC and a chronic myeloid leukemia (CML) tumor cell model; and (iii) verify if those RNAs are packaged into EVs shed by the NSCLC and CML MDR cells. To accomplish this, we performed an analysis of the drug response on sensitive cells following co-culture with EVs released by MDR cells and analyzed the RNA profiles, by next generation sequencing, of drug-sensitive cells and their MDR counterparts, as well as of EVs released by those cells. To assess if those RNAs were of a specific tumor type, two different tumor models were studied: NSCLC and CML.

## 2. Results

### 2.1. Extracellular Vesicles Released by MDR Cells Transfer Drug-Resistance to Recipient Drug-Sensitive Cells

**Figure 1:**
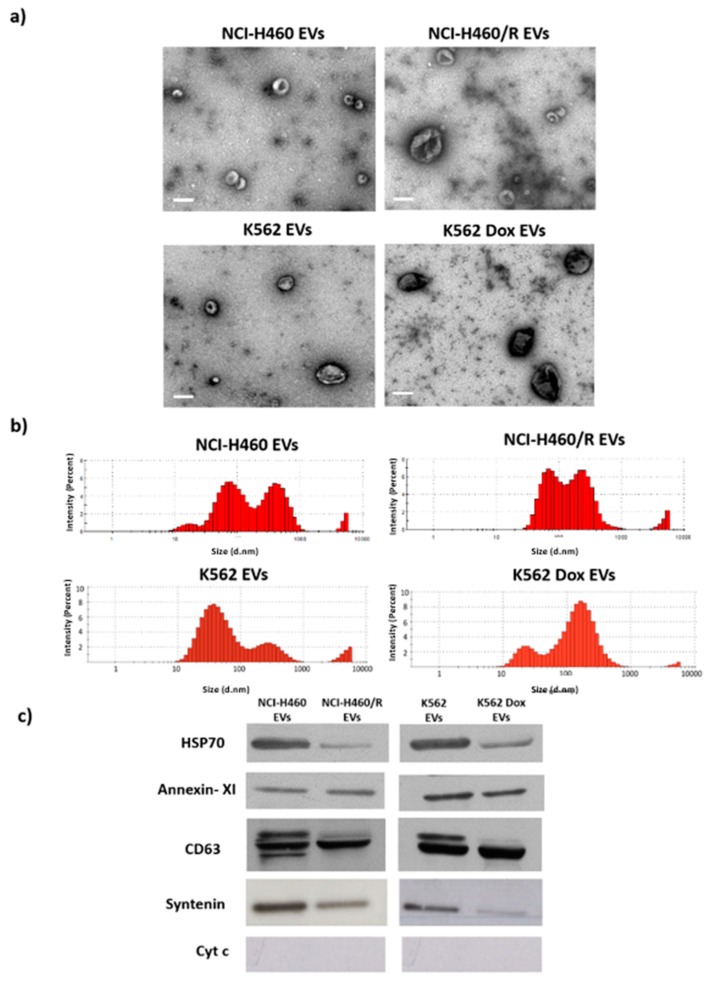
Characterization of extracellular vesicles (EVs) isolated from pairs of drug-sensitive and multidrug resistance (MDR cells), from non-small cell lung cancer (NSCLC) and chronic myeloid leukemia (CML) cell lines (NCI-H460/ NCI-H460/R and K562/K562Dox, respectively). (**a**) Morphology of EVs, analyzed by TEM. Bar corresponds to 200 nm. (**b**) EVs’ size distribution, analyzed by dynamic light scattering (DLS). (**c**) Levels of HSP70, Annexin XI, CD63, syntenin, and cytochrome in EVs, analyzed by Western blotting.

**Figure 2:**
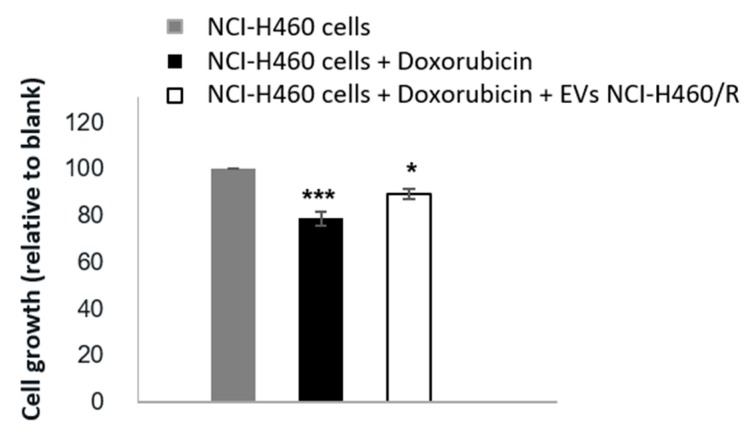
Effect of EVs released by MDR (NCI-H460/R) cells from NSCLC on the response of drug-sensitive cells (NCI-H460) to doxorubicin treatment. Cell growth was determined on drug-sensitive cells (grey bar), and drug-sensitive cells co-cultured without (black bar) or with (white bar) EVs released by MDR cells using the sulphorhodamine B (SRB) assay. Results are the mean ± S.E. of three independent experiments. * *p* < 0.05; *** *p* < 0.001 (NCI-H460 cells without drug treatment vs. with doxorubicin treatment).

In our previous studies, EVs from drug-sensitive and MDR cells were isolated using the same methodology as described in the present study (differential ultracentrifugation) and thoroughly characterized [19,20,21], presenting an average size range from 50 to 150 nm and classical EV markers (Hsp70, Syntenin, and CD63). Some of those features were further confirmed in the EVs isolated in this study (Figure 1). In the present work, to confirm that EVs released by MDR cells are able to transfer a drug-resistant phenotype to drug-sensitive cells, as previously described by others [22,23,24], NCI-H460 (drug-sensitive) cells were co-cultured with EVs isolated from the NCI-H460/R (MDR) cells and then treated with doxorubicin. The results (Figure 2 and Supplementary Figure S1) show that drug-sensitive cells, when previously incubated with EVs from MDR cells, became less sensitive to doxorubicin. We would like to mention that the uptake of MDR-EVs by drug-sensitive cells was confirmed (unpublished data from a manuscript currently under revision [25]). Importantly, the MDR phenotype of the NCI-H460/R cells was confirmed and previously shown by us [19]. Furthermore, the presence of P-gp in both MDR cell lines was also previously demonstrated [19].

### 2.2. Differences Were Observed between the Profile of Small RNAs from Cells and EVs

In order to identify non-coding RNAs associated with MDR, a small RNA profile was analyzed in two pairs of MDR and drug-sensitive counterparts: one pair from NSCLC and the other pair from CML. In addition, to investigate if those non-coding RNAs were packaged into the cargo of the EVs released by those cells, a small RNA profile was analyzed in the two corresponding pairs of EVs.

**Figure 3:**
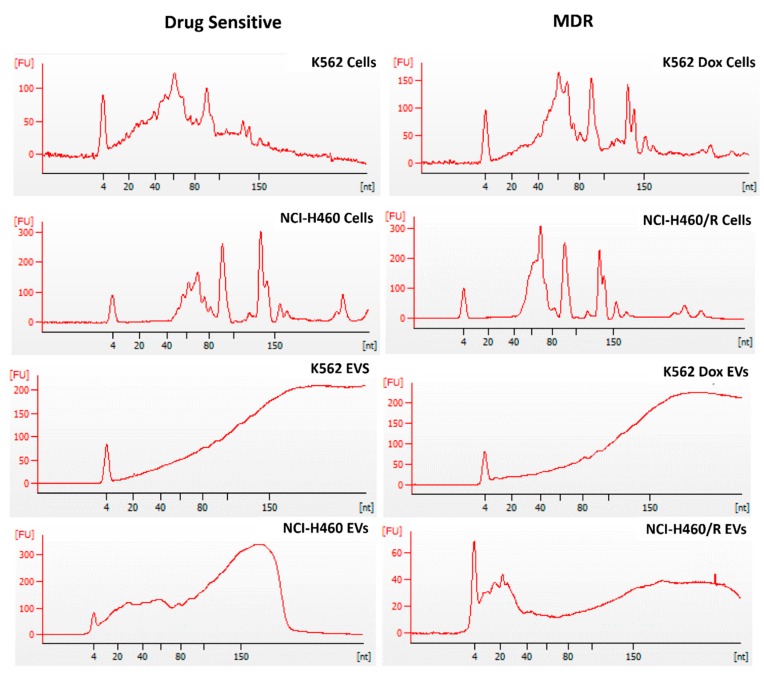
Profile of small RNAs from drug-sensitive and MDR counterpart cells and from the EVs released by those cells. Cells were from two models: NSCLC (NCI-H460 and NCI-H460/R) and CML (K562 and K562Dox). RNA was analyzed using the Small RNA chip of the Bioanalyzer 2100 Agilent. Images are representative of three independent biological replicates.

The cellular small RNAs were more heterogeneous regarding the size range, with the tRNA peak being observed in all cell samples (~66 nt) (Figure 3). In contrast, in EVs, this peak was not evident. Interestingly, the small RNA profiles of the EVs from the two tumor models (NSCLC and CML) were different. Indeed, it was verified that EVs released from the NSCLC cells had greater amounts of small RNA species within the range of 20–40 nt than the EVs released from the CML cells. In both tumor models, a peak around 150 nt was present, possibly corresponding to ribosomal RNA 5.8S or small nuclear RNA [26,27].

### 2.3. RNA Deep Sequencing Showed Several Classes of Transcripts in Cells and in EVs Released by Those Cells

To further determine the identity of the small RNA molecules, next generation sequencing (NGS) was performed. Deep sequencing results were checked using FastQC and all 24 samples passed the test. Following this, alignment with the human genome was performed. Between 70% and 95% successful alignment between RNA reads and HG19 was observed (Supplementary Table S1), even though only moderate coverage was obtained in the case of EVs.

**Figure 4:**
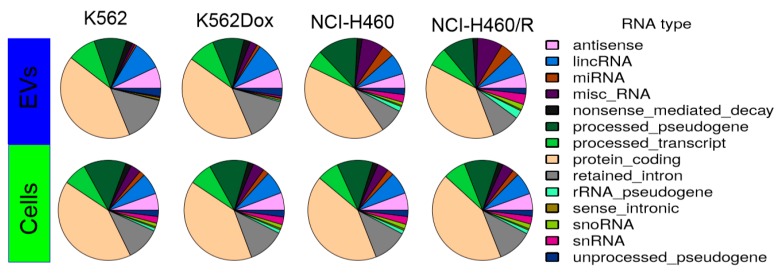
Composition of the mapped reads of RNAs in cells and EVs. Results were obtained by using ENSEMBL transcript annotation.

A pie chart of the distribution of mapped reads showed a similar composition of RNA from cells and from EVs released by those cells (Figure 4). Among non-coding RNAs, high levels of pseudogenes were found in all conditions (13–17%). Moreover, in agreement with the Bioanalyzer profile, EVs released by NSCLC cells showed higher levels of miRs, when compared with EVs released by CML cells. Importantly, multiple dimensional scaling analysis and principal component analysis revealed a cluster between the independent replicates in all conditions, indicating proper reproducibility. Furthermore, the two independent clusters observed for drug-sensitive vs. MDR conditions, in cells and EVs from both tumor models, suggest that the results have biological relevance (Supplementary Figures S2a,b and S3a,b).

### 2.4. Selective Package of RNAs in the Cargo of EVs Released by MDR Cells

**Figure 5:**
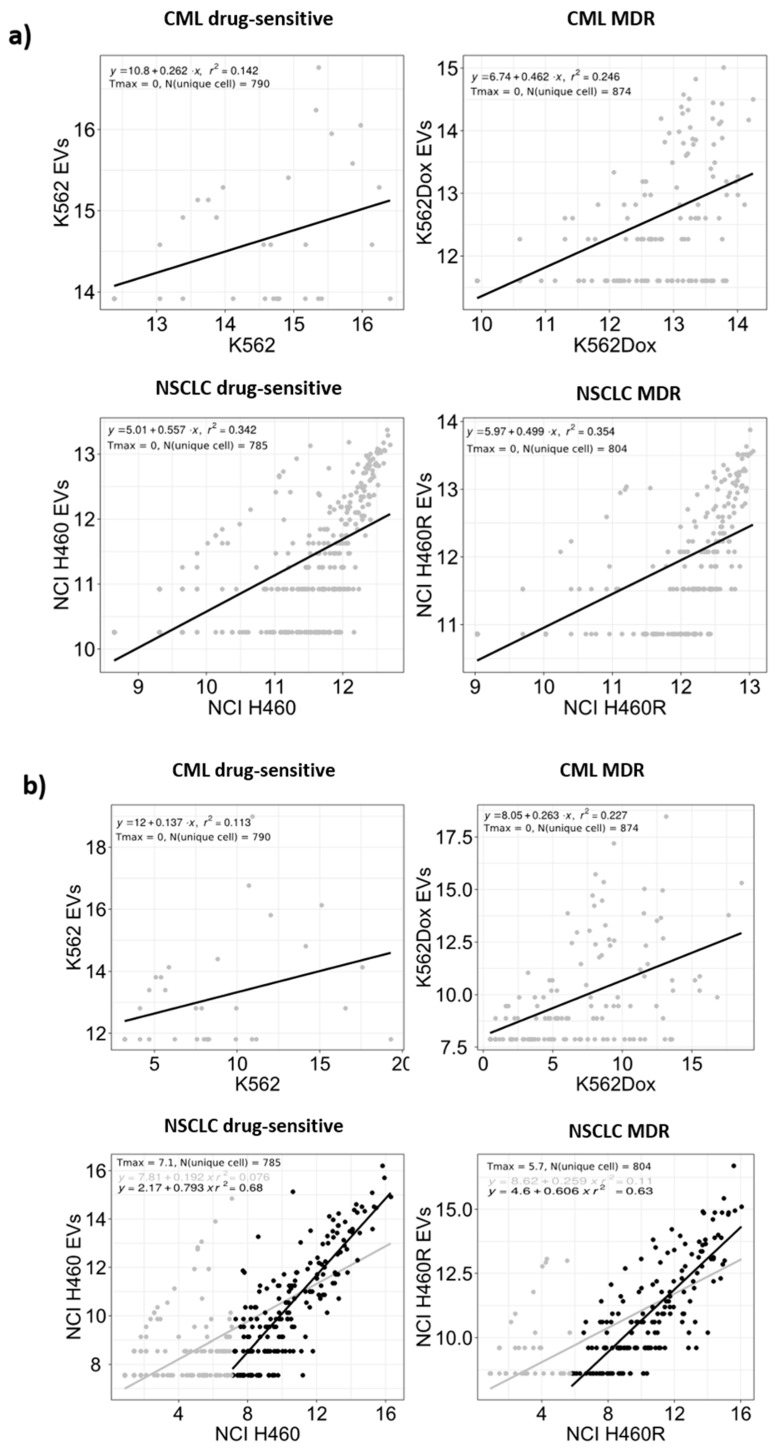
RNA species (**a**) and microRNAs (miRs) (**b**) log2 (RPM) abundance in the extracellular vesicles versus their donor cells. Values are presented on a log2 scale and summarize the results from three independent biological replicates. Tmax indicates the optimized threshold value and N (unique cell) the number of RNAs or miRs with counts in cells, but not in EVs.

To analyze if RNAs present in EVs reflect the intracellular RNAs, analysis of linear regression plots (log2 reads) was performed for the sequenced reads in EVs and cells (for each condition). All RNA species present in EVs were also present in cells (as expected), in both tumor models. In contrast, EVs did not harbor all RNA species present in cells. The same observation was found regarding the miRs content. Figure 5 summarizes the comparison of RNA species in EVs versus donor cells, for RNA species present in both EVs and donor cells. Linear regressions were performed for all log2 RPM values above zero. In addition, a linear grid search to find the noise threshold that yielded the highest R squared value was performed from 0 to 10, with 0.1 increases per step on the log2 RPM scale. The regression line for the optimized threshold is not depicted in Figure 5 for the cases where the optimized R squared value was insignificantly different from that obtained using a zero threshold. We observed a better correlation between EVs and donor cells for the NSCLC model when compared with the CML model. Furthermore, the R squared value for miRs was approximately twice the R squared value for RNAs in the NSCLC model. Nevertheless, the obtained *r* squared value was around 0.6, indicating no direct correlation between species present in EVs and species present in cells.

### 2.5. Significant Regulated RNA Species in MDR Cells and/or EVs

Next, the differences between the data sequenced for drug-sensitive and MDR cells and from the EVs released by those cells were assessed. First, using ENSEMBL annotation for several RNA species with FeatureCounts software, aligned reads corresponding to ribosomal RNA, tRNA, snRNA, or pseudogenes were identified. These results were further analyzed by the R package EdgeR, which allowed a pairwise comparison of drug-sensitive vs. MDR for each of the cell models, cells, and EVs. RNA species presenting *p*-values < 0.05 in each comparison were considered differently expressed. The relative expression of each entity in each comparison is shown in Supplementary Tables S2–S9.

**Figure 6:**
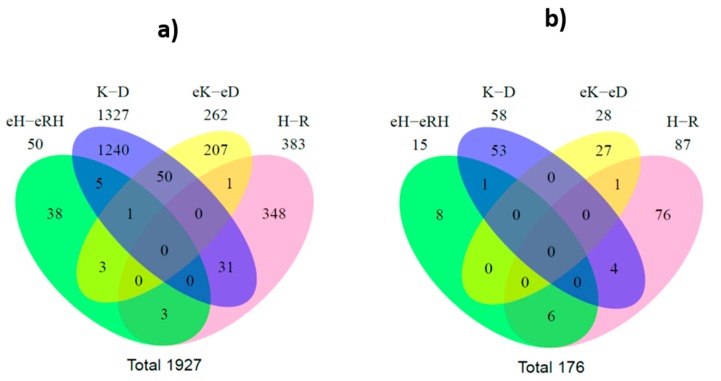
Venn diagram of significantly differentially regulated RNA species (**A**) and miRs (**B**). eH: NCI-H460 EVs; eRH: NCI-H460/R EVs; eK: K562 EVs; eD: K562Dox EVs; H: NCI-H460 cells; R:NCI-H460/R cells; K-K562 cells; D: K562Dox cells.

Moreover, Venn diagrams were generated based on differences between drug-sensitive and MDR cells and also on differences between the EVs released by those cells. A total of 1927 differently expressed RNA species were found (*p* < 0.05) (Figure 6a).

Regarding the analysis of cells, 31 RNA species were found to be altered between drug-sensitive cells and their MDR counterparts, in both tumor models. From these, three transcripts (miR-383, miR-4660, and chromosome 16 open reading frame 45) were identically found to be overexpressed in MDR cells of both tumor models (NSCLC and CML). In addition, two other transcripts (serglycin and FOXF1 adjacent non-coding developmental regulatory RNA) were identically found to be downregulated in MDR cells of both tumor models (NSCLC and CML, Table 1).

Regarding the analysis of EVs, three pseudogenes were found to have altered levels between drug-sensitive and MDR EVs, in both tumor models. From these, two had higher levels in the MDR EVs, which are annotated as a novel pseudogene and RNA 5.8S ribosomal pseudogene 2 in Table 1. To validate the alignment of reads of these three pseudogenes, the regions from the alignment were extracted using samtools [28] and manually visualized.

When comparing the results from cells and EVs in the CML model, fifty species were found with different levels in both the cells and EVs, between MDR and drug-sensitive counterparts. In the NSCLC model, three RNA species were found with different levels in both the cells and EVs, between MDR and drug-sensitive counterparts. No RNA species was found to be differently expressed in both tumor models between MDR cells and their drug-sensitive counterparts and simultaneously on their EVs.

A parallel analysis using an annotation for miRs with FeatureCounts software was also performed. As described for RNA species analysis, the results from EdgeR software overlapped in a Venn diagram. A total of 176 differentially expressed miRs were found (Figure 6b). Interestingly, four miRs (miR-383-5p, miR-335-5p, miR-504-5p, and miR-933) were found to be differently expressed between drug-sensitive and MDR cells, in both CML and NSCLC models. From these, the ones found to be similarly increased or decreased in NSCLC and CML MDR cells are shown in Table 1.

Moreover, six miRs (miR-204-5p, miR-139-5p, miR-29c-5p, miR-551b-3p, miR-29b-2-5p, and miR-204-3p) had simultaneously altered levels in both cells and EVs from NSCLC.

**Figure 7:**
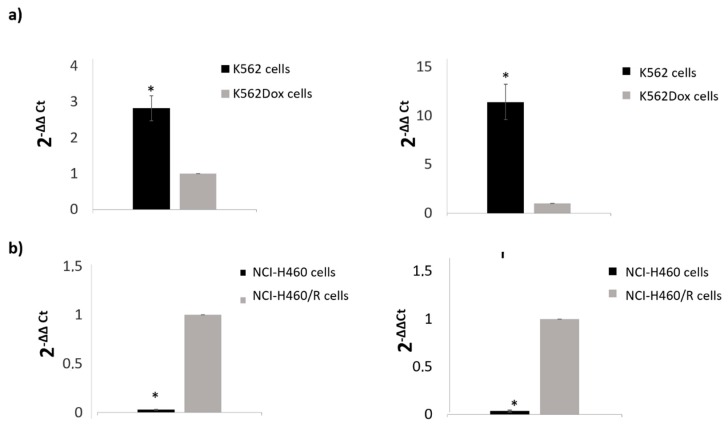
Validation by qRT-PCR of miRs found altered in deep sequencing analysis in CML (**a**) and NSCLC (**b**) models. (**a**) right panel: miR-99a-5p and left panel: miR-335-5p; (**b**) right panel: miR-335-5p and left panel: miR-204-5p. The YY-axis represents the fold-change of selected genes obtained by real-time PCR (ΔΔCt method) relative to MDR cells. Results are expressed as the mean ± SEM.

To validate some of the results obtained, two miRs found altered and highly abundant for each tumor model were selected to be analyzed by quantitative real-time PCR (qRT-PCR). The results (Figure 7) confirmed the previous ones obtained with NGS and bioinformatics analysis.

### 2.6. Functional Analysis of the Targeted Genes

**Figure 8:**
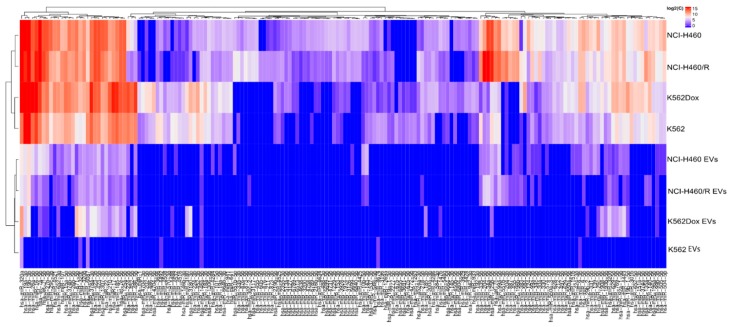
Hierarchical clustering of significant regulated miRs in drug-sensitive and MDR counterpart cells and their respective EVs.

To infer the biological relevance of the previous findings, two approaches were used. Firstly, a hierarchical clustering of all significantly regulated miRs was performed. In both models, drug-sensitive cells and MDR counterparts were clustered together, as expected (Figure 8). Similar results were observed for the EVs (Figure 8). Importantly, the differential expression of some miRs was observed between drug-sensitive and MDR conditions in both tumor models, in agreement with the results from the Venn diagram.

Moreover, a functional analysis of the possible targeted mRNAs of the miRs found altered by the Venn diagram analysis was performed, using DAVID bioinformatics tools. The results indicated, as expected, that the deregulated pathways between drug-sensitive and MDR conditions (cells and EVs, in both tumor models) are greatly associated with cancer (Supplementary Figure S4). The molecular function analysis indicated that the targeted mRNAs are involved in regulation of the transcriptome and proteome (Supplementary Figure S5).

## 3. Discussion

MDR is one of the main limitations of cancer treatment efficacy. MDR results from a complex network of altered pathways that allows malignant cells to proliferate without responding to chemotherapy [1]. In order to overcome MDR in patients, it is essential to understand the multifactorial and complex nature of MDR, including the triggers and mechanisms involved in the development and dissemination of this phenotype. In recent years, the possibility that EVs released by MDR cells may contribute to drug resistance in recipient drug-sensitive cells has been described [4]. In addition, it was found that drug-efflux pumps (such as P-gp), as well as non-coding RNAs (namely microRNAs and long non-coding RNAs), are present in the cargo of EVs released by drug-resistant cells [29], possibly contributing to the horizontal transfer of drug resistance to recipient cells [4]. Nonetheless, the majority of these studies were carried out with a probe-based methodology [30]. Therefore, using high throughput technology, which allows the analysis of a great amount of data (including previously undescribed species), in this work, we aimed to analyze the RNA species from pairs of MDR and drug-sensitive cells and from the EVs released by those cells. We studied two different tumor models: CML and NSCLC. For each model, a pair of cell lines was used, consisting of a drug-sensitive cell line and its MDR (P-gp overexpressing) counterpart.

To the best of our knowledge, this is the first study assembling data obtained by deep sequencing from pairs of drug-sensitive and MDR counterpart cells, as well as from their EVs. In our previous studies, EVs from drug-sensitive and MDR cells have been isolated using the same methodology as described in the present study and thoroughly characterized [19,20,21]. Some of those features were further confirmed in the EVs isolated in this study. In the present work, we confirmed the evidence obtained by other authors [22,23,24] on the ability of EVs released by MDR cells to transfer a drug-resistant phenotype to drug-sensitive recipient cells. Furthermore, in order to identify important players of this MDR phenotype, we analyzed the RNA content of both cells and EVs.

Our results showed that MDR cells and their drug-sensitive counterparts have different contents of RNA species. Moreover, the EVs released by MDR cells also have different RNA entities from the EVs released by their drug-sensitive counterparts. Importantly, following EV isolation, treatment with proteinase K and RNase was performed before RNA extraction, strengthening the association of extracellular RNA results to EVs and not to protein-RNA precipitates.

First, we observed that the Bioanalyzer profile of small RNA molecules is different between cells and EVs, as previously shown by other authors [31]. Usually, the ncRNAs are named according to their size: small RNAs (which include short ncRNAs with 17–32 bp (miRNAs, piRNAs, and tiRNAs)), mid-size ncRNAs with 60–200 bp (snoRNAs, PASRs, TSSs-RNAs, and PROMPTs), and long ncRNAs with more than 200 bp (lincRNAs, T-UCRs, and other long ncRNAs) [32]. In our models, the type of RNA species was, in general, similar between cells and EVs. Importantly, pseudogenes were the most abundant RNA species present in all samples, ranging from 13% to 17% of abundance, with miRs representing only approximately 0.6–4.3% of the total small RNAs. This value seems lower than expected, but another study showed that miRs may be a minor form of RNAs in EV cargo, whereas tRNA fragments and Y-RNAs were described as being specifically secreted into EVs (by the donor cells) [33]. In contrast, another study showed that even though EVs contain proportionally less small RNAs than cells, their small RNA fraction was enriched in miRs [34]. Therefore, the relative abundance of miRs in EV cargo is still under debate and it might be influenced by pathophysiological changes [35]. In the present study, a relatively higher abundance of miRs (compared to total RNA species) was found in EVs released by NSCLC cells than in those cells. Nevertheless, this finding was not confirmed in the CML model, indicating that the relative abundance of miRs could also be dependent of the tumor type.

Deep sequencing tools have revealed that the small RNA species found in cells go far beyond the classical small interfering, piwi-associated, and miR families. Non-protein-coding functional RNAs such as rRNA, tRNA, snRNA, and snoRNA also have a role, or at least a predictive role, in pathway regulation [33]. Our results showed differential levels of some RNA species between drug-sensitive and MDR conditions (cells and EVs). To better comprehend the data, a Venn diagram allowed the interception of the four groups (drug-sensitive cells vs. MDR cells; drug-sensitive EVs vs. MDR EVs, from both NSCLC and CML models). A total of 1927 RNA species were found to be altered between drug-sensitive and MDR conditions. Importantly, three species were found to be altered in the EVs from both models. Interestingly, these three species were all pseudogenes. This result is in agreement with the pie chart results showing the distribution of mapped reads, where there was a higher percentage of pseudogenes. Two of these pseudogenes were increased in EVs released by MDR cells from both tumor models: a novel pseudogene and the RNA5.8S ribosomal pseudogene 2. Pseudogenes are genes that have lost their ability to synthesize proteins due to events such as premature stop codons, splicing errors, and frameshift-causing deletions or insertions [36]. The diagnostic and prognostic value of pseudogenes in cancer has been described. Some studies have associated alterations in pseudogenes with overall survival or disease-free survival [37]. Three important examples are *PTENP1*, *E2F3P1*, and *OCT4-pg1* in renal cell carcinoma [38], hepatocellular carcinoma [39], and gastric cancer [40], respectively. Interestingly, the presence of pseudogenes in EVs was previously found. Indeed, pseudogenes associated with ribosomal proteins or heat-shock proteins, among others, were previously detected in other studies, as confirmed in the Vesiclepedia (a database of EV content [41]). However, in the majority of those studies, the presence of pseudogenes was not discussed. Therefore, the role of pseudogenes in cells and EVs needs further clarification. We argue that the two pseudogenes found to be increased in EVs released by MDR cells (from NSCLC and CML) in this study may be further studied regarding their possible application as MDR-related biomarkers in blood plasma. Importantly, five transcripts were also found to be similarly altered (increased or decreased) in MDR cells from both models: miR-383, miR-4660, chromosome 16 open reading frame 45, FOXF1 adjacent non-coding developmental regulatory RNA, and serglycin. These transcripts may also be further investigated as putative MDR-biomarkers. Interestingly, four of these transcripts were previously associated with the regulation of tumor cell proliferation [42], epithelial-mesenchymal transition [43], stemness [43], metastization [43,44,45], poor prognosis [44], and poor survival [46].

There are different triggering factors for MDR traits, some of which are tumor-specific mechanisms (e.g., genetic alterations). Notably, as mentioned above, in the present work, a hematological (CML) and a non-hematological (NSCLC) tumor model were studied. The cellular features of both models are very different, but both the MDR cell lines have the overexpression of P-gp as the main mechanism responsible for their MDR phenotype in common. Therefore, the study of these two different tumor MDR models may allow the identification of alterations associated with an MDR phenotype caused by (or responsible for) P-gp overexpression. Importantly, in the hierarchical clustering of miRs, each pair of cell lines (drug-sensitive and MDR) clustered together, suggesting that the drug-resistant phenotype has a smaller effect on miRs regulation than the source of the cell line. The same result was observed for EVs.

The specific differential expression of miRs between drug-sensitive and MDR (cells and EVs) was also analyzed by a Venn diagram. The obtained results showed a panel of six miRs with different levels between drug-sensitive and MDR cells and EVs in the NSCLC model: miR-204-5p, miR-139-5p, miR-29c-5p, miR-551b-3p, miR-29b-2-5p, and miR-204-3p. Therefore, we suggest that these miRs should be further studied as potential MDR biomarkers of NSCLC. Indeed, all these miRs were previously associated with tumor cell proliferation or cancer progression [47,48,49,50,51]. Importantly, the differential expression of miR-204-5p was further validated by qRT-PCR. Further analysis of the Venn diagram allowed the identification of two miRs with altered levels between drug-sensitive and MDR cells, in both tumor models: miR-383-5p and miR-504-5p. Therefore, we suggest that they may have a putative role in P-gp-associated MDR. To the best of our knowledge, the regulation of P-gp by those miRs has never been described. Importantly, both miRs were previously associated with the regulation of tumor cell proliferation and cancer drug resistance [42,52,53]. It is well-known that one single miR has different cellular targets and thus, depending on the cellular context, the same miR may have tumor-suppressor or tumor-promotion properties. Therefore, the miR relative levels described above should be interpreted taking into account the alterations observed (decrease or increase in the MDR condition, for each model). For example, published studies by two independent groups have shown that miR-451 and miR-27a were upregulated in MDR tumor cell lines and caused an increase in P-gp levels [54,55]. However, other studies have described the opposite effect for these two miRs, regarding the effect on P-gp expression and drug resistance [56,57]. These apparently contradictory results may be explained by the different cellular contexts in the different studies [58]. Importantly, using the miRTarBase, the targets of miR-383-5p and miR-504-5p were predicted. Interestingly, one of the targets found, TP53INP1, was previously studied in a drug-resistant context. Indeed, the features of a drug-resistant breast cancer cell line were assessed by genomic approaches and *MDR1* overexpression, together with miRNA-mediated TP53INP1 down-regulation, were found [59]. In addition, two other predicted targets of those miRs, NCKAP1 and E2F7, were previously associated with metastization and drug resistance, respectively [60,61].

The NGS results were validated by a qRT-PCR analysis of miRs found to be significantly regulated and highly abundant. The levels of miR-99a, miR-204-5p, and miR-335-5p were quantified. Importantly, the alterations observed between drug-sensitive and MDR conditions were concordant with the alterations obtained by NGS analysis. Interestingly, evidence of the involvement of those miRs in tumor proliferation and cancer drug resistance was previously shown. MiR-204-5p (together with miR-211-5p) was associated with resistance to a BRAF inhibitor in melanoma cells [51]. In addition, miR-99a-5p was shown to be a tumor-suppressor miR, by targeting mTOR in human urinary bladder urothelial carcinoma cells [62] and miR-335-5p regulation was associated with tumor proliferation and invasion [63].

The mechanisms postulated for the sorting of small RNAs (particularly miRs) into EVs were elegantly reviewed by R. Boheme et al. [64]. Briefly, miRs sorting is regulated by (i) RISC (RNA-induced silencing complex)-associated proteins, (ii) cellular miRs/target mRNA levels, (iii) 3′ non-template terminal nucleotide additions, (iv) 3′ sequence motifs/protein guides, and (v) ceramide biosynthesis. Nonetheless, the selection of EV cargo by the donor cells is still under extensive study and not fully understood. Some authors have described that the sorting of RNA species into EVs is selective, while other authors have defended a non-selective packaging of this cargo [33,65]. In our models, some species were more abundant in EVs then in origin cells. Tosar J.P. et al. described that when intracellular expression values for individual miRs reached a certain threshold, a direct correlation between intracellular and extracellular values was observed, suggesting a non-selective packaging of miRs into EVs. In our results, a linear grid search to find the noise threshold that yielded the highest R squared value was performed from 0 to 10, with 0.1 increases per step on the log2 RPM scale. Interestingly, regardless of how the thresholds were set, we did not obtain R squared values above 0.8, as described by those authors. In addition, a number of data points were viewed to be far from the optimized regression line. Therefore, we can conclude that the data for the CML and NSCLC models presented in this study supports the sorting/selective packing of some miRs into EVs to some extent. Indeed, from the Venn diagram analysis of either the RNA species or the miRs in particular, some RNA entities were found to be differentially present, between MDR and drug-sensitive models, only in the EVs and not in the cells, in both tumor models. Moreover, the pie chart analysis (of the NSCLC model) showed a higher percentage of miRs in EVs when compared with cells. Taken together, these results suggest that some RNA species are probably selectively packaged into EVs.

## 4. Materials and Methods

### 4.1. Cell Culture

Two pairs of cell lines from two different human tumor models were used, consisting of a drug-sensitive cell line (DS) and its MDR (P-gp overexpressing) counterpart: (i) NCI-H460 (DS) and NCI-H460/R (MDR) non-small cell lung cancer cells (NSCLC) (a kind gift of Dr. M. Pešić, Belgrade, Serbia [66,67]) and (ii) K562 (DS) and K562Dox (MDR) chronic myeloid leukemia cells (CML) (a kind gift of Dr. J.P. Marie, Paris, France [68,69]). To maintain the MDR phenotype, 100 nM doxorubicin was added to the NCI-H460/R cells every month and 1 μM doxorubicin was added to the K562Dox cells every 2 weeks. All cell lines were genotyped and routinely monitored for possible mycoplasma contamination by PCR (Cell Culture and Genotyping Service, i3S). Cells were cultured in RPMI-1640 medium with Ultraglutamine I and 25 mM HEPES (Biowest) supplemented with 10% fetal bovine serum (FBS, Biowest) at 37 °C in a humidified incubator with 5% CO_2_ in air. Experiments were performed with cells exhibiting exponential growth and over 90% viability.

### 4.2. EV Isolation

Cells were grown in conditioned media consisting of RPMI with 10% EV-depleted FBS (previously ultracentrifuged at 4 °C for at least 16 h). Cell culture medium was collected and differentially centrifuged as described by Thery et al. [70]. Briefly, the sequence of centrifugations was as follows: 300 g for 10 min; 2000 g for 10 min; 10,000 g for 30 min; and 100,000 g for 1 h 15 min. The final pellet was washed in PBS. For RNA downstream analysis, the pellet was re-suspended in PBS and stored at −80 °C. For co-culture studies, the pellet was re-suspended in EV-depleted medium and immediately used.

### 4.3. Electron Microscopy

EVs re-suspended in PBS were adsorbed onto Formvar-carbon coated electron microscopy grids at room temperature for 2 min. Following this, EVs were stained with 5% uranyl acetate and visualized with a JEM 1400 transmission electron microscope at an accelerating voltage of 80 kV (Jeol, Tokyo, Japan).

### 4.4. Dynamic Light Scattering

The EV size/diameter was measured by dynamic light scattering (DLS), using a Nano series Malvern Zetasizer Instrument (Prager Elektronik, Wolkersdorf im Weinviertel, Austria). Measurements were carried out at 20 °C, at 633 nm, and the back scattered light was recorded at an angle of 173°. The mean hydrodynamic diameter of exosomes was calculated by fitting a Gaussian function to the measured size distribution. Three measurements per sample were performed.

### 4.5. Western-Blotting

EVs were lysed in Winman’s buffer (1% NP-40, 0.1 M Tris–HCl pH 8.0, 0.15 M NaCl, and 5 mM EDTA [Ethylenediaminetetraacetic acid]) with EDTA-free protease inhibitor cocktail (Roche, Basel, Switzerland), and quantified using a modified Lowry assay (Bio-Rad, Hercules, CA, USA). After quantification, 5 μg of proteins was separated on a 12% Bis–Tris SDS-PAGE gel and transferred into a nitrocellulose membrane (GE Healthcare, Cleveland, OH, USA). Membranes were then incubated with the anti-HSP70, anti-annexin-XI, anti-CD63, anti-syntenin, and anti-cytochrome c primary antibody. Signals were detected using the ECL Western blot Detection.

### 4.6. Co-Culture of EVs with Cells

NCI-H460 cells were seeded in 96-well plates (5000 cells/well). After 24 h, the EVs (isolated from 80 mL of conditioned medium, which corresponds to around 10 µg/well) from NCI-H460/R cells were added to the sensitive cells (NCI-H460) and 24 h later, treatment with 50 nM doxorubicin (Sigma-Aldrich^®^, St. Louis, MO, USA) was performed.

### 4.7. Sulphorhodamine B Assay

Following 24 h of co-culture, the sulphorhodamine B (SRB) assay was carried out as previously described [71]. Briefly, cells were fixed with 10% (w/v) ice cold trichloroacetic acid (TCA), washed with distilled water, and incubated with 0.4% (w/v) SRB for 30 min. After washing with 1% acetic acid, 10 mM Tris base was used to solubilize the SRB. Absorbance was measured at 510 nm using a multiplate reader (Synergy Mx, Biotek Instruments Inc., Winooski, VT, USA), as previously described [72].

### 4.8. RNA Extraction

The isolated EVs were treated with proteinase K (0.05 µg/µL) and RNase (0.5 µg/µL) [73]. RNA from cells and EVs was extracted using the same kit: miRCURY™ RNA Isolation Kit–Cell & Plant (Exiqon, Foster City, CA, USA), following the manufacturer’s instructions. RNA quality was evaluated using Bioanalyzer (Agilent 2100, Santa Clara, CA, USA). Samples (from three independent biological replicates) were stored at −80 °C until library preparation.

### 4.9. Library Preparation and Next Generation Sequencing

Libraries were constructed according to the Ion Total RNA-Seq Kit v2 protocol (Life Technologies, Carlsbad, CA, USA), with modifications reported by Cheng et al. [74]. Briefly, 3 μL of total RNA samples were incubated for 16 h with provided reagents for adaptors’ hybridization and ligation. After incubation, reverse transcription was performed and cDNA was purified and size-selected with magnetic beads. Afterwards, cDNA was amplified with specific primers containing different barcodes (BC) for sample identification and tracking. A second round of purification and size-selection was performed with magnetic beads. Library evaluation and quantification was assessed with TapeStation 2200. Each sample was ligated to a unique barcode (BC) for sample identification and tracking. Due to the presence of adapter dimers in some of the samples, an adapted protocol was developed, consisting of the running of the final libraries into a 4% agarose gel and band excision, thus avoiding the adapter dimer bands. A second evaluation was performed and the results were compared: libraries in the range of the typical size distribution were selected, diluted to 80 pM, and pooled. The pooled libraries were further processed on the Ion Chef™ System (Life Technologies, Carlsbad, CA, USA)) and the resulting 550™ chips were sequenced on the Ion S5™XL System (Life Technologies, Carlsbad, CA, USA) [74].

### 4.10. Bioinformatics and Statistical Analysis

A total of 24 FASTQ files from the Ion Torrent sequencer were analyzed. Files were trimmed using CutAdapt with the following parameters: the minimum fragment length was set to 10 and minimum quality to 20 [75]. The quality of the trimmed reads was assessed by FASTQC (Andrews S. Fast QC, A Quality Control tool for High Throughput Sequence Data, 2014; [76]). Reads were then aligned by Bowtie1 to human genome assembly (hg19), allowing for one mismatch. Prebuild indexes were downloaded from the Bowtie webpage. The length of seed substrings in bowtie analysis was set to 19 and the additional parameters—best –nomaqround were also specified. The read count was calculated using FeatureCounts [77] software implemented in miARma-Seq [78] using a minimum quality of 10. The genome annotation files were miRBase_Annotation_20_for_hsa_mature_miRNA.gtf and/Homo_sapiens.GRCh37.75.gtf for miRNAs and all RNAs, respectively. Significant differential expression was calculated using the EdgeR [79] minimum count per million cut-off of 2. biomaRt [80] was used to retrieve transcript annotation. Multiple discriminant analysis (MDS) plots were made with the R package Limma using the minimum count per million cut-off of 2 [81]. Target mRNAs were determined by extracting significantly regulated miRNA after the correction of multiple testing (*p* value < 0.05) and mapping targets using miRTarBase [82]. Linear regression plots were made in R using the ggplot package. For the linear regression plots, raw read count data summed across the three independent replicas were transformed to reads per million (RPM) values filtered to only contain RNA species detected in both of the conditions that were compared. Next, the values were log2 transformed. A noise threshold was defined by optimizing linear correlation by evaluating *r* squared values of the regression models. The optimization was performed by a linear grid search from 0 to 10 on the log2 RPM scale. For each comparison, linear regression was performed for all data points and for the threshold-filtered data using the optimal threshold found by the linear grid search. GO and KEGG functional analysis were performed by DAVID [83] using false discovery rate (FDR)-corrected *p* values.

### 4.11. NGS Validation by qRT-PCR

For both tumor models, two miRs for each model were selected for further validation of the NGS results, conducted by quantitative real time PCR (qRT-PCR). The total RNA (2 ng) was reverse transcribed using the miScript II Reverse Transcription Kit (Qiagen, Hilden, Germany), according to the manufacturer’s instructions. The miRNA expression levels were then measured using the miSCRIPT Primer Assay and the Quantitect SYBR Green PCR Kit (Qiagen), according to the manufacturer’s instructions. Dissociation curves were generated. The relative expression ratio for each miR was normalized to another miR found to be abundantly expressed and whose expression levels did not differ between drug-sensitive and MDR conditions in both NSCLC and CML models (miR-19b-3p). Data was analyzed with the 2^−ΔΔCt^ method [84].

## 5. Conclusions

In the present work, using high throughput technology, we were able to compare the RNA species present in drug-sensitive and MDR counterpart cells and in the EVs released by those cells. Importantly, two pseudogenes (a novel pseudogene and RNA 5.8S ribosomal pseudogene 2) were found to be upregulated in EVs released by MDR cells, in both tumor models studied (NSCLC and CML). In addition, a panel comprised of miR-204-5p, miR-139-5p, miR-29c-5p, miR-551b-3p, miR-29b-2-5p, and miR-204-3p was also found to be upregulated in EVs released by MDR cells and in their donor MDR NCSLC cells. Therefore, these pseudogenes and miRs should be further studied as potential biomarkers of MDR in cancer and further studies need to be carried out to confirm their role in MDR.

## Figures and Tables

**Table 1:** Transcripts found altered in MDR cells or in EVs shed by MDR cells, with identical regulation in NSCLC and CML tumor models. Log FC above > 1 and *p* value < 0.05 indicate upregulation in the MDR condition.

			NSCLC		CML	
	Transcript Name	Ensembl_Transcript_ID	Log FC	*p*-Value	Log FC	*p*-Value
**Cells**	**miR-383**	ENST00000362257	−8.83	0.0000	-5.46	0.0000
	**miR-4660**	ENST00000583549	−2.88	0.0221	−4.09	0.0318
	**chromosome 16 open reading frame 45**	ENST00000565913	−1.77	0.0272	−2.77	0.0455
	**serglycin**	ENST00000242465	2.05	0.0297	5.69	0.0035
	**FOXF1 adjacent non−coding developmental regulatory RNA**	ENST00000599749	3.85	0.0346	3.01	0.0274
	**miR−504−5p**	ENST00000385065	−1.38	0.0041	−1.74	0.0054
**EVs**	**novel pseudogene**	ENST00000311910	2.480	0.0246	4.50	0.0068
	**RNA, 5.8S ribosomal pseudogene 2**	ENST00000363564	1.81	0.0291	3.57	0.0006

## Supplementary Materials

The following are available online at https://www.mdpi.com/2072-6694/12/1/200/s1, Figure S1: Effect of EVs released by MDR cells on the viable cell number of drug-sensitive recipient cells treated with doxorrubicin, in the CML model. Figure S2: Multiple dimensional scaling using read counts of all mapped RNA transcripts (a) and all mapped miRs (b) as input. Figure S3: Principal Component Analysis using read counts per million of all mapped RNA transcripts (a) and all mapped miRs (b) as input. Figure S4: Functional analysis of the possible target mRNAs of the miRs found significantly altered. The functional analyses were performed by DAVID. Figure S5: Molecular function of the possible target mRNAs of the significant regulated miRs (obtained by Venn diagram analysis and extracted targets mRNAs) were obtained from miRTarBase [82]. Table S1: Total number of sequenced reads, number of processed reads after FASTQ quality control, number of alignments mapped to human genome (HG19) and reads obtained in FeatureCounts (RNA species) for each biological replicate of drug-sensitive and MDR cells, as well as respective EVs, in both CML (K562,K562Dox) and NSCLC (NCI-H460,NCI-H460/R) cell models. Table S2: Log Fold Change of RNA species expression between drug-sensitive (NCI-H460) cells and their MDR (NCI-H460/R) counterparts. Log FC above > 1 and *p* value < 0.05 indicate up regulation in MDR condition. Table S3: Log Fold Change of RNA species expression between drug-sensitive (K562) cells and their MDR (K562Dox) counterparts. Log FC above > 1 and *p* value < 0.05 indicate up regulation in MDR condition. Table S4: Log Fold Change of RNA species expression between EVs released by drug-sensitive (NCI-H460) cells and the EVs released by their MDR (NCI-H460/R) counterparts. Log FC above > 1 and *p* value < 0.05 indicate up regulation in MDR condition. Table S5: Log Fold Change of levels of RNA species between EVs released by drug-sensitive (K562) cells and EVs released by their MDR (K562Dox) counterparts. Log FC above > 1 and *p* value < 0.05 indicate up regulation in MDR condition. Table S6: Log Fold Change of levels of miRs between drug-sensitive (NCI-H460) cells and their MDR (NCI-H460/R) counterparts. Log FC above > 1 and *p* value < 0.05 indicate up regulation in MDR condition. Table S7: Log Fold Change of miRs expression between drug-sensitive (K562) cells and their MDR (K562Dox) counterparts. Log FC above > 1 and *p* value < 0.05 indicate up regulation in MDR condition. Table S8: Log Fold Change of levels of miRs between EVs released by drug-sensitive (NCI-H460) cells and EVs released by their MDR (NCI-H460/R) counterparts. Log FC above > 1 and *p* value < 0.05 indicate up regulation in MDR condition. Table S9: Log Fold Change of miRs expression between drug-sensitive (K562) cells and EVs derived from their MDR (K562Dox) counterparts. Log FC above > 1 and *p* value < 0.05 indicate up regulation in MDR condition.
